# Aquaporin-8 overexpression is involved in vascular structure and function changes in placentas of gestational diabetes mellitus patients

**DOI:** 10.1515/biol-2022-0522

**Published:** 2022-11-14

**Authors:** Yanxing Shan, Jiawen Cui, Xinyi Kang, Weichun Tang, Yiling Lu, Ying Gao, Liping Chen

**Affiliations:** Department of Obstetrics and Gynecology, Affiliated Hospital 2 of Nantong University, No. 6 North Road, Haierxiang, Chongchuan District, Nantong, Jiangsu, 226001, China; Department of Obstetrics and Gynecology, Qingpu Branch of Zhongshan Hospital Affiliated to Fudan University, Qingpu, Shanghai, 201700, China

**Keywords:** gestational diabetes mellitus, AQP8, placentas vessels, endothelial cells

## Abstract

To study the role and mechanism of aquaporin-8 (AQP8) in placental vascular development in gestational diabetes mellitus (GDM), hematoxylin–eosin staining and immunohistochemistry were utilized to analyze the histopathological changes in placentas in GDM patients. Transwell, CCK-8, and tube formation assays were performed to examine cell migration, proliferation, and tube formation. AQP8, vascular cell adhesion molecule 1 (VCAM-1), tumor necrosis factor alpha (TNF)-α, and vascular endothelial growth factor (VEGF)-A expression levels were investigated. Relative to the control group, the placentas in the GDM group showed morphological changes, the number of microvessels in the placental villi arterioles was significantly higher, and the area of microvessels in the arterioles of placental villi was significantly lower. The expression levels of VCAM-1, TNF-α, VEGF-A, and AQP8 in the GDM placentas and human umbilical vein endothelial cells (HUVECs) stimulated by high glucose were significantly higher than those in the control group, and AQP8 was located in placental endothelial cells. Overexpression of glucose and AQP8 inhibited tube formation, migration, and proliferation in HUVECs. High glucose levels can induce dysfunction in vascular endothelial cells and lead to pathological changes in the placental vascular structure in GDM. AQP8 overexpression in placental GDM can inhibit endothelial cell behavior, cause endothelial cell dysfunction, and further participate in the occurrence and development of GDM placental vascular lesions.

## 1 Introduction

Gestational diabetes mellitus (GDM) is a gestational disease that occurs during pregnancy, and it accounts for 16.9% of the global prevalence of gestational hyperglycemia [1,2]. It is one of the most common obstetric diseases and seriously endangers the health of the mother and child, and it can cause premature rupture of membranes, macrosomia, intrauterine growth retardation, neonatal hypoglycemia, and other complications [3,4,5,6], and it can even have long-term adverse effects on the health of the mother and child [7]. In 2019, on the basis of 51 studies performed in 41 countries, the International Diabetes Federation reported an incidence rate of hyperglycemia in 15.8% of pregnant women aged 20–49 years, with 83.6% of the cases due to GDM [8]. The prevalence of GDM has been reported to be high worldwide at 6.5–25.1% [9]. The incidence rate of GDM in China has markedly increased over the last few decades [10]. Currently, treatments for GDM are relatively limited [11,12], which is mainly because the etiology and mechanism of GDM remain unclear. Therefore, exploring the pathogenesis of GDM, understanding the degree of disease progression, and identifying methods of reducing the occurrence of GDM from its source are still urgent problems to be solved.

Placentas are important organs for material exchange between the mother and fetus during pregnancy [13]. The normal development of placental vessels is important for fetal survival and growth, and angiogenesis disorders can cause many pregnancy-related diseases. The abnormal development of placental vessels is a key factor in the occurrence and development of GDM [14]. Huynh et al. reported that the terminal villus volume, surface area, capillary volume, and length of the placenta in pregnant women with diabetes were significantly greater than those in healthy pregnant women [15]. The placental villi of pregnant women with GDM are dysplastic and accompanied by abnormal chorionic branches and excessive neovascularization. Sáez et al. showed that placental vascular endothelial cell damage is an important feature of placental vascular lesions and revealed that dysfunction of vascular endothelial cells provides the pathological basis for placental vascular lesions in GDM [16]. These structural and functional abnormalities in the GDM placenta may be associated with adverse pregnancy outcomes. Therefore, the behavior of placental vascular endothelial cells in GDM must be further investigated to understand the pathogenesis of adverse pregnancy outcomes.

Aquaporins (AQPs) are a group of glycoproteins with similar structures and functions and are widely distributed in tissues and cells [17,18]. Previous studies have suggested that AQPs mainly mediate the passive transport of water molecules, which maintains the water balance inside and outside the cell [19]. However, recent studies have shown that AQPs can be detected in vascular endothelial cells and may also participate in angiogenesis, cell migration, adhesion, and other biological processes [20]. Hua et al. found that knockout of AQP1 in mice reduced tumor angiogenesis [21]. Koun et al. found that aquaporin-8 (AQP8) is expressed in the blood vessels of zebrafish and participates in early angiogenesis [22]. All of the aforementioned studies have indicated that AQPs play an important role in the regulation of angiogenesis; however, the exact mechanism is unknown.

Previous studies have found that AQP8 is localized on vascular endothelial cells, and AQP8 is highly expressed in GDM placentas [23,24]. Thus, we proposed a scientific hypothesis that the high expression of AQP8 in GDM placentas may be involved in changes in the vascular structure and the function in GDM placentas. This study aimed to reveal the role and the mechanism of AQP8 in placental vascular pathological changes associated with GDM. These findings not only contribute to the further understanding of the pathogenesis of placental vasculopathy in GDM but also provide new ideas for the clinical diagnosis and treatment of GDM and judgment of prognosis.

## 2 Materials and methods

### 2.1 Study subjects and tissue samples

Thirty pregnant women with GDM who delivered at the Second Affiliated Hospital of Nantong University between January 2019 and September 2020 were recruited for this study. The inclusion criteria were as follows: (1) Han nationality; (2) single birth; (3) gestational weeks at delivery: 37–42 weeks; (4) age: 22–34 years; (5) number of pregnancies ≤3; (6) body mass index (BMI) before pregnancy: 19–24 kg/m^2^; (7) satisfactory blood glucose control during pregnancy after diet, exercise, or insulin therapy; and (8) no systemic diseases. Patients with the following conditions were excluded: (1) severe hepatic and renal insufficiency; (2) cardiovascular and cerebrovascular diseases; (3) pregnancy-related diseases such as pregnancy-induced hypertension, thyroid disease, placenta previa, and placental abruption; (4) history of mental illness; (5) history of alcohol and drug abuse; (6) negative health habits such as smoking; (7) antiphospholipid syndrome; and (8) history of serious infectious diseases. Thirty healthy pregnant women were randomly assigned to the control group. No congenital malformations were found in any group, and no obvious abnormalities were found in the umbilical cord and placentas such as the prepositional placenta, racket-shaped placenta, sail placenta, excessively short or long cord, and true knot of the umbilical cord. Within 5 min after delivery of the placentas, the decidual layer on the surface of the maternal decidua was removed, and placental tissues (1 cm × 1 cm × 1 cm) were taken from the placental center (placental lobule of the umbilical cord attachment area). The samples were cleaned, packed, numbered with sterile sodium chloride solution, stored in liquid nitrogen for 30 min, and transferred to a –80°C refrigerator.


**Informed consent:** Informed consent has been obtained from all individuals included in this study.
**Ethical approval:** The research related to human use has been complied with all the relevant national regulations, institutional policies and in accordance with the tenets of the Helsinki Declaration, and has been approved by the Medical Ethics Committee of the Second Affiliated Hospital of Nantong University.

### 2.2 Hematoxylin–eosin (HE) staining

After collecting all the placenta samples, they were fixed in 10% buffered formalin for 24 h and then dehydrated by washing under an ascending gradient of ethanol. The samples were then sectioned and embedded in paraffin wax for HE staining. The number and the area of microvessels were counted, and the diameter of intervillous vessels was measured randomly in five obvious staining visions of placental sections using the nanoZoomer digital pathology (NDP) tool at 20× magnification under a microscope (Nikon Ts2, Japan). Placental tissue staining was performed by a senior pathologist.

### 2.3 Immunohistochemistry

The expression of CD31 and AQP8 in the placental samples was detected by immunohistochemistry, as previously described [25]. Briefly, the sections were incubated with primary antibodies (AQP8 (1:100, Novus Biologicals) and CD31 (1:50, Abcam)) overnight at 4°C. The sections were then incubated with MaxVision-HRP (1:5,000, Martianit Technology Co., Ltd.) for 2 h at 37°C. Subsequently, images were taken at 100× magnification under a microscope (Nikon Ts2, Japan). The vessel area was measured using the NDP tool.

### 2.4 Cell culture and transfection

Human umbilical vein endothelial cells (HUVECs) were obtained from the Department of Otolaryngology, Affiliated Hospital of Nantong University, which were inoculated into a six-well plate (cell density was approximately 30%), and medium containing 10% fetal bovine serum was added for culturing (without antibiotics). When the cells grew to approximately 70% confluency, the culture medium was removed from the pore plate, and serum-free medium (without antibiotics) was added. Simultaneously, the transfection reagent was slowly added and cultured at 37°C under 5% CO_2_ for 4–6 h. They were then replaced with complete medium (10% fetal bovine serum + 1% double antibody) and cultured for 24–48 h. According to the instructions of Lipofectamine 2000, the transfection reagent (5 μL pcDNA3.1 plasmid containing the *AQP8* gene or siRNA targeting AQP8 was fused with 250 μL of 5 mmol/L d-glucose dulbecco’s modified eagle medium (DMEM) and incubated at room temperature for 20 min) was added and transfected.

### 2.5 Scratch assay

Three horizontal lines were drawn on the bottom of a six-well plate using a marker pen, and the cells were digested with trypsin and inoculated into six-well plates. The cells were cultured at 37°C under 5% CO_2_ for 24–48 h, and images were captured under a microscope (Nikon Ts2, Japan).

### 2.6 Transwell assay

High-glucose stimulated/transfected cells were digested with trypsin, and the concentration was adjusted to 3 × 10^4^/mL. Then, 700 μL of medium containing DMEM and 5 mmol/L d-glucose + 5% fetal bovine serum was added to the 24-hole plate. Transwell migration assay was conducted as previously described [26]. Cells were observed and counted under a microscope (Nikon Ts2, Tokyo, Japan). All experiments were performed in triplicate.

### 2.7 Cell counting kit-8 (CCK-8) assay

The cells were counted using a CCK-8 kit, and the results were measured using a microplate reader (Infinite M100 PRO, Tecan, Männedorf, Switzerland) at a wavelength of 450 nm. The experiments were repeated at least three times.

### 2.8 Tube formation assay

Tube formation assay was conducted as previously described [27]. After culturing the cells in a 37°C incubator for 2, 4, and 6 h, images were acquired using a microscope (Nikon Ts2, Japan). Each experiment was performed in triplicate.

### 2.9 Reverse transcription-quantitative polymerase chain reaction (RT-qPCR)

Total RNA was isolated using (TRIzol) RNAiso Plus reagent (TaKaRa, Kyoto, Japan). RNA (1 µg) was reverse-transcribed using the PrimeScript™ RT Master Mix (TaKaRa). The purity and concentration were detected using a NanoDrop. RT-qPCR was conducted using Power SYBR Green PCR Master Mix (Thermo, NY, USA). A standard amplification protocol was used according to the manufacturer’s instructions. GAPDH was used as a reference gene. The primers used are listed in Table 1.

**Table 1:** Primers for real-time fluorescent quantitative PCR detection

Primers		Sequence (5′–3′)
AQP8	Forward	TCCTGAGGAGAGGTTCTGGA
	Resverse	AGGGCCCTTTGTCTTCTCAT
VCAM-1	Forward	TGTTGAGATCTCCCCTGGAC
	Resverse	GAATTGGTCCCCTCACTCCT
VCAM-A	Forward	CCCACTGAGGAGTCCAACAT
	Resverse	AAATGCTTTCTCCGCTCTGA
TNF-α	Forward	GTCAACCTCCTCTCTGCCAT
	Resverse	CCAAAGTAGACCTGCCCAGA
GAPDH	Forward	CAGCCTCAAGATCATCAGCA
	Resverse	GGATCTCGCTCCTGGAAGATG

### 2.10 Western blot

The cells were lysed, and total proteins were collected. Total protein (20 μg) was added per lane, separated by sodium dodecyl sulfate-polyacrylamide gel electrophoresis, and transferred onto a polyvinylidene fluoride membrane (Millipore, USA). After blocking with 5% skim milk in tris buffered saline and tween. for 2 h at room temperature, the membranes were incubated at 4°C overnight with primary antibodies, including AQP8 (1:1,000, Novus Biologicals) and GAPDH (1:10,000, Abcam). Following incubation with goat antirabbit antibody and goat antimouse antibody (1:5,000, Jackson ImmunoResearch Inc.) for 2 h at room temperature, proteins on the membrane were visualized with a chemiluminescence kit (Thermo Scientific, USA) and analyzed using ImageJ software.

### 2.11 Statistical analysis

The obtained data were analyzed using the GraphPad Prism software (version 8.0). Statistical analyses were performed using SPSS 22.0. Quantitative variables that conformed to a normal distribution were analyzed based on the means ± standard deviation ( \bar{x}] ± S). Differences between two or more groups were compared using Student’s *t*-test and one-way analysis of variance. The results were considered statistically significant only when *P* < 0.05.

## 3 Results

### 3.1 Clinical characteristics of patients

Among the subjects, one case of premature membrane rupture and six cases of perineum laceration occurred in the healthy pregnant women, while two cases of gestational hypertension, four cases of preeclampsia, five cases of premature rupture of membranes, four cases of premature delivery, two cases of oligohydramnios, one case of polyhydramnios, seven cases of perineum laceration, one case of fetal distress, seven cases of macrosomia, and one case of fetal growth restriction occurred in the pregnant women with GDM. As shown in Table 2, significant differences were not observed between the GDM and control groups in terms of maternal age, gestational age, BMI before pregnancy, weight gain during pregnancy, birth weight, or infant sex (all *P* > 0.05).

**Table 2:** Clinical characteristics of the study population

Characteristic	Control (*n* = 30)	GDM (*n* = 30)	*P*-values
Maternal age (years)	29.1 ± 3.1	29.4 ± 2.8	0.740
Gestational age (weeks)	39.1 ± 0.8	38.8 ± 0.8	0.160
BMI before pregnancy (kg/m^2^)	22.0 ± 1.6	22.2 ± 1.2	0.748
Weight gain during pregnancy (kg)	14.3 ± 1.9	12.9 ± 3.9	0.032
Fasting plasma glucose (FPG; mmol/L)	4.6 ± 0.3	5.2 ± 0.6	<0.0001
One hour plasma glucose (1hPBG, mmol/L)	7.9 ± 1.1	9.2 ± 1.9	0.0015
Two hour plasma glucose (2hPBG; mmol/L)	6.5 ± 1.1	8.4 ± 1.8	<0.0001
Gestational hypertension, % (*n*)	0	2 (6.7%)	0.492
Preeclampsia, % (*n*)	0	4 (13.3%)	0.112
Premature rupture of membranes, % (*n*)	1 (3.3%)	5 (16.7%)	0.195
Preterm birth, % (*n*)	0	4 (13.3%)	0.112
Oligohydramnios, % (*n*)	0	2 (6.7%)	0.492
Polyhydramnios, % (*n*)	0	1 (3.3%)	>0.999
Perineal laceration, % (*n*)	6 (20.0%)	7 (23.3%)	>0.999
Fetal distress, % (*n*)	0	1 (3.3%)	>0.999
Macrosomia, % (*n*)	0	7 (23.3%)	0.011
Fetal growth restriction, % (*n*)	0	1 (3.3%)	>0.999

Values are mean ± SD.

### 3.2 Histopathology

**Figure 1:**
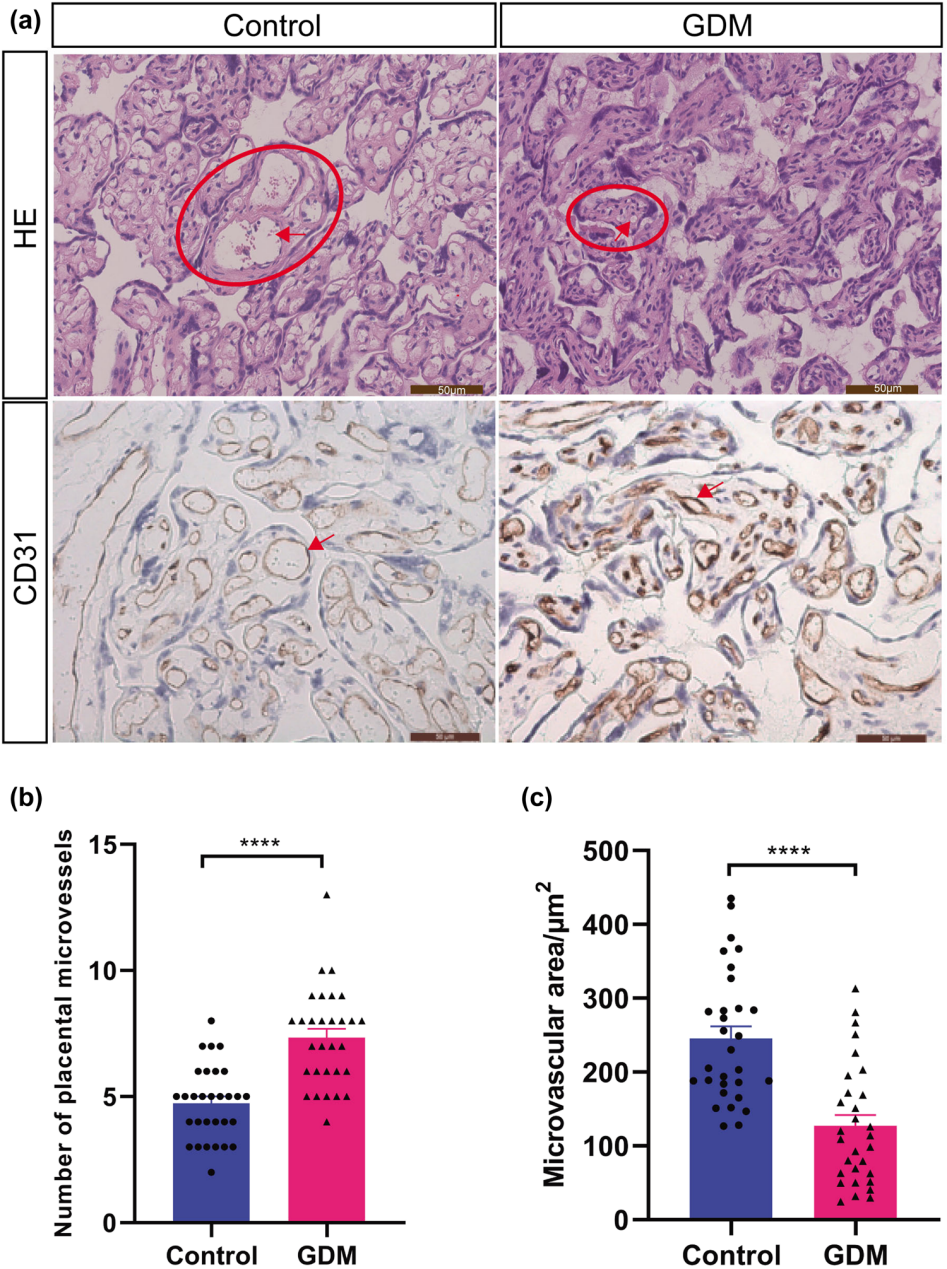
Pathological changes in the placenta of GDM patients. (a) HE staining and immunohistochemistry revealed pathological changes in placenta tissues (20×). (b) Number of microvessels in the arterioles of the villi. (c) Area of microvessels in the arterioles of placental villi. **** *P* < 0.0001. The circle in the figure shows villous stems, and the arrow points to the villous arteriole.

As shown in Figure 1a, when compared with the control group, the placentas in the GDM group showed morphological changes, such as thickening of the arteriole wall, stenosis of the lumen, and poor maturation of villi. The number of microvessels in the placental villus arterioles in the GDM group was significantly higher than that in the control group (*P* < 0.05; Figure 1b). The area of microvessels in the arterioles of placental villi in the GDM group was significantly lower than that in the control group (*P* < 0.05; Figure 1c).

### 3.3 High expression of VCAM-1, TNF-α, VEGF-A, and AQP8 in GDM placentas

**Figure 2:**
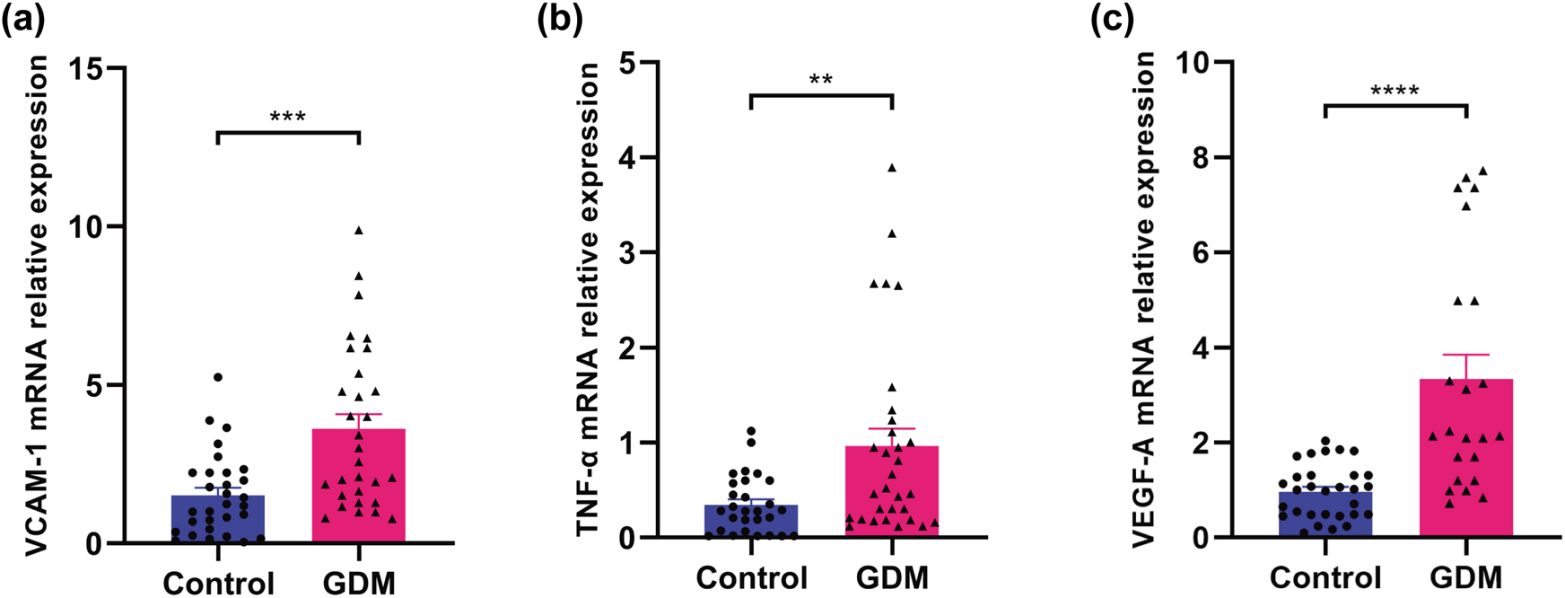
mRNA expression of VCAM-1, TNF-α, and VEGF-A in the placenta. (a) VCAM-1 mRNA expression level. (b) TNF-α mRNA expression level. (c) VEGF-A mRNA expression level. ** *P* < 0.01, *** *P* < 0.001, and **** *P* < 0.0001.

**Figure 3:**
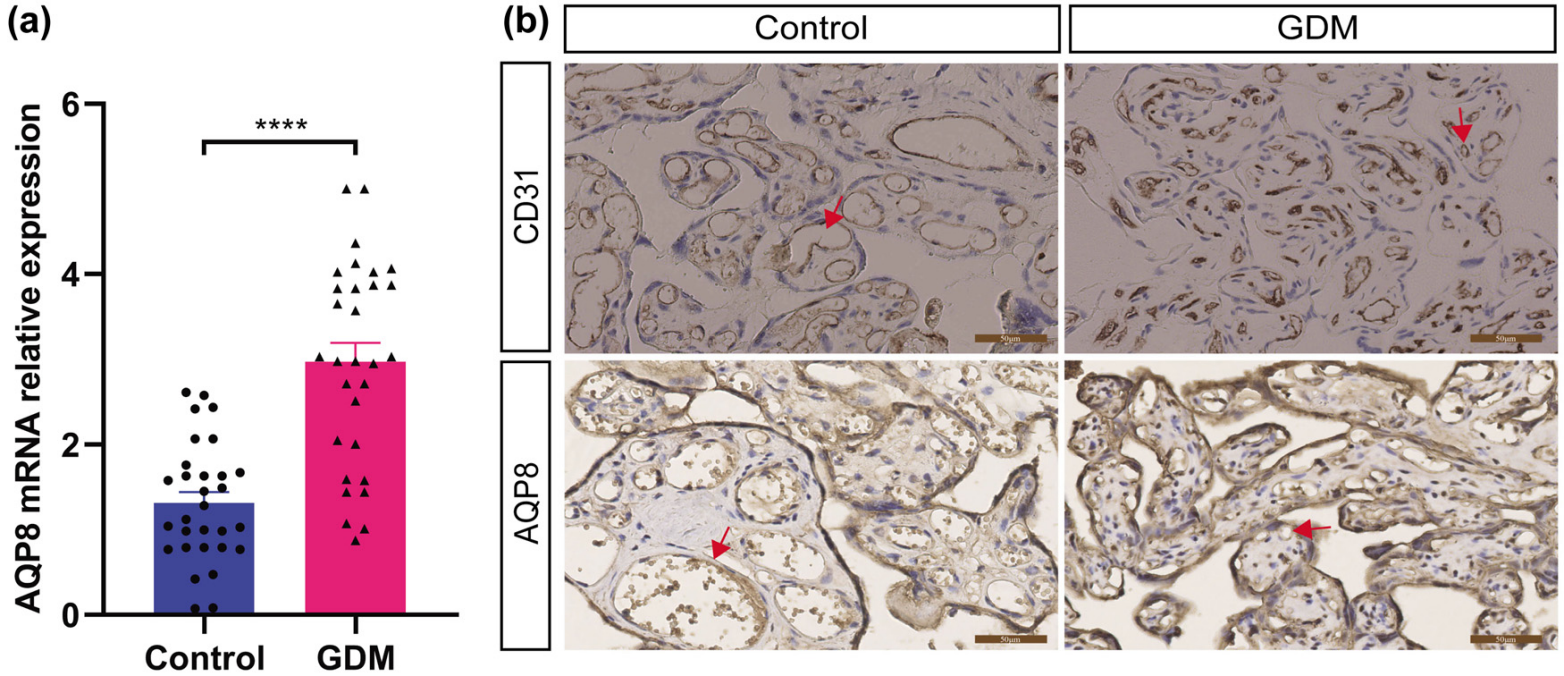
Location and mRNA expression level of AQP8 in the placenta (100×). (a) The mRNA expression level of AQP8 in GDM placentas. (b) AQP8 located in placental endothelial cells. **** *P* < 0.0001. The circle in the figure shows the villous stem, and the arrow points to the villous arteriole.

Evidence has shown that vascular cell adhesion molecule 1 (VCAM-1), tumor necrosis factor alpha (TNF-α), and VEGF-A are closely related to vascular lesions, especially VCAM-1, which is a biomarker of endothelial dysfunction [28,29,30]. In this study, the results in Figure 2 showed that the expression levels of VCAM-1, TNF-α, and VEGF-A in GDM placentas were significantly higher than those in the control group (all *P* < 0.05). In addition, evidence also has shown that AQP8 was detected in vascular endothelial cells and is involved in angiogenesis, cell migration, adhesion, and other biological processes [24]. Here, the location and expression levels of AQP8 were determined, and the results showed that the mRNA expression level of AQP8 in GDM placentas was significantly higher than that in the control group (*P* < 0.05; Figure 3a). AQP8 was located in placental endothelial cells after immunolabeling with the CD31 antibody (specific labeling of vascular endothelial cells) and AQP8 antibody (Figure 3b).

### 3.4 High glucose promoted the expression of VCAM-1, TNF-α, VEGF-A, and AQP8 in HUVEC

**Figure 4:**
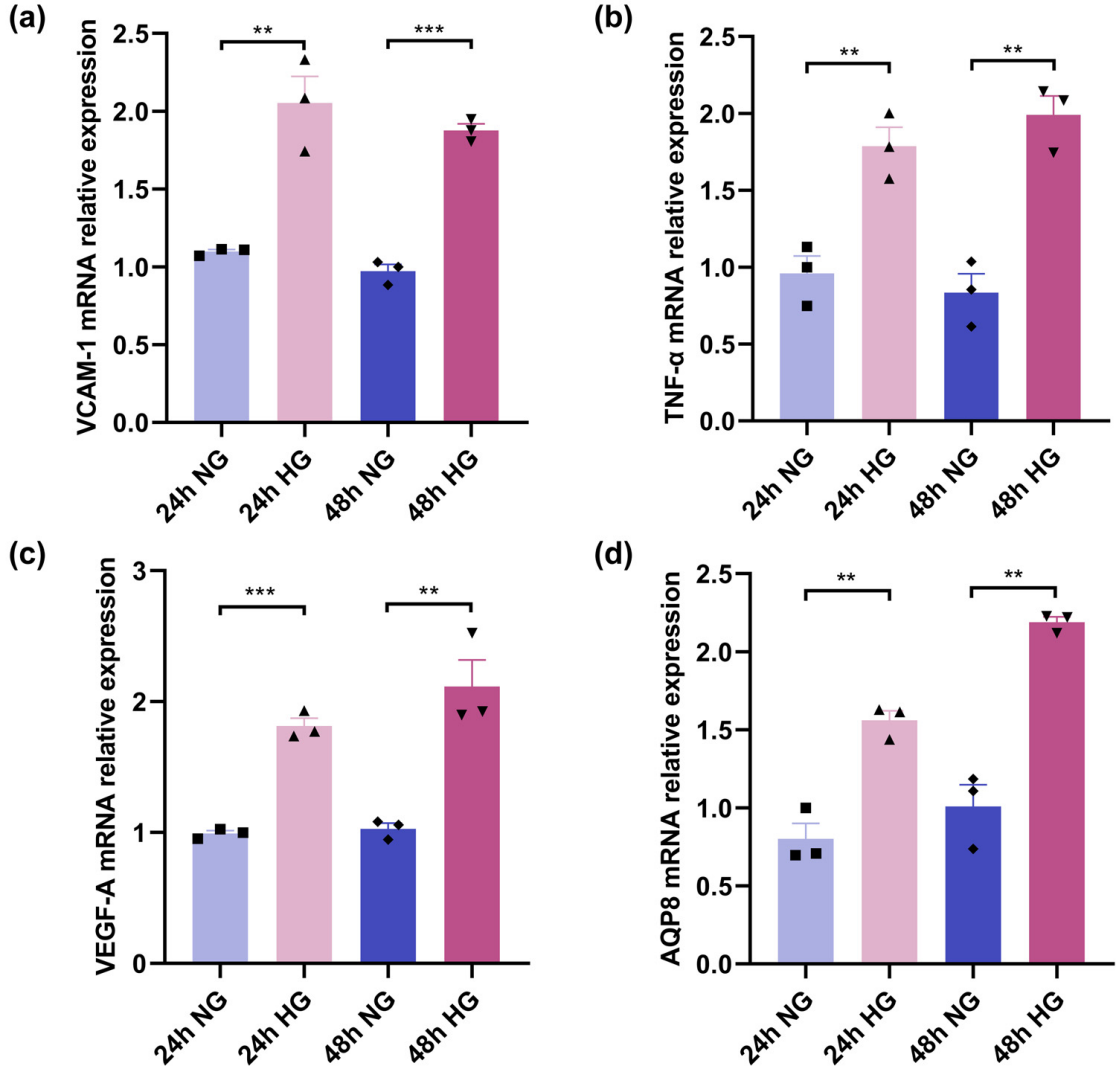
mRNA expression levels of VCAM-1, TNF-α, VEGF-A, and AQP8 in HUVECs after high glucose stimulation. (a) VCAM-1 mRNA expression level. (b) TNF-α mRNA expression level. (c) VEGF-A mRNA expression level. (d) AQP8 mRNA expression level. ** *P* < 0.01, *** *P* < 0.001. HG represents the high glucose stimulation group; NG represents the normal glucose concentration group.

AQP8 expression was found in GDM placental vascular endothelium by immunohistochemistry; thus, HUVECs were selected as the cell line to detect the effect of AQP8 on vascular endothelial behavior. HUVECs were stimulated with 30 mmol/L d-glucose to establish a high-glucose injury model, as described previously [31,32,33]. The expression levels of VCAM-1, TNF-α, VEGF-A, and AQP8 were detected after HUVECs were stimulated with high glucose. The results showed that the mRNA expression levels of VCAM-1, VEGF-A, and TNF-α significantly increased in HUVECs stimulated by high glucose for 24 and 48 h (all *P* < 0.05; Figure 4a–c). After high glucose stimulation for 24 and 48 h, the mRNA expression level of AQP8 in the HUVECs was significantly higher than that in the normal group (all *P* < 0.05; Figure 4d).

### 3.5 High glucose inhibited the ability of tube formation, migration, and proliferation of HUVEC cells

**Figure 5:**
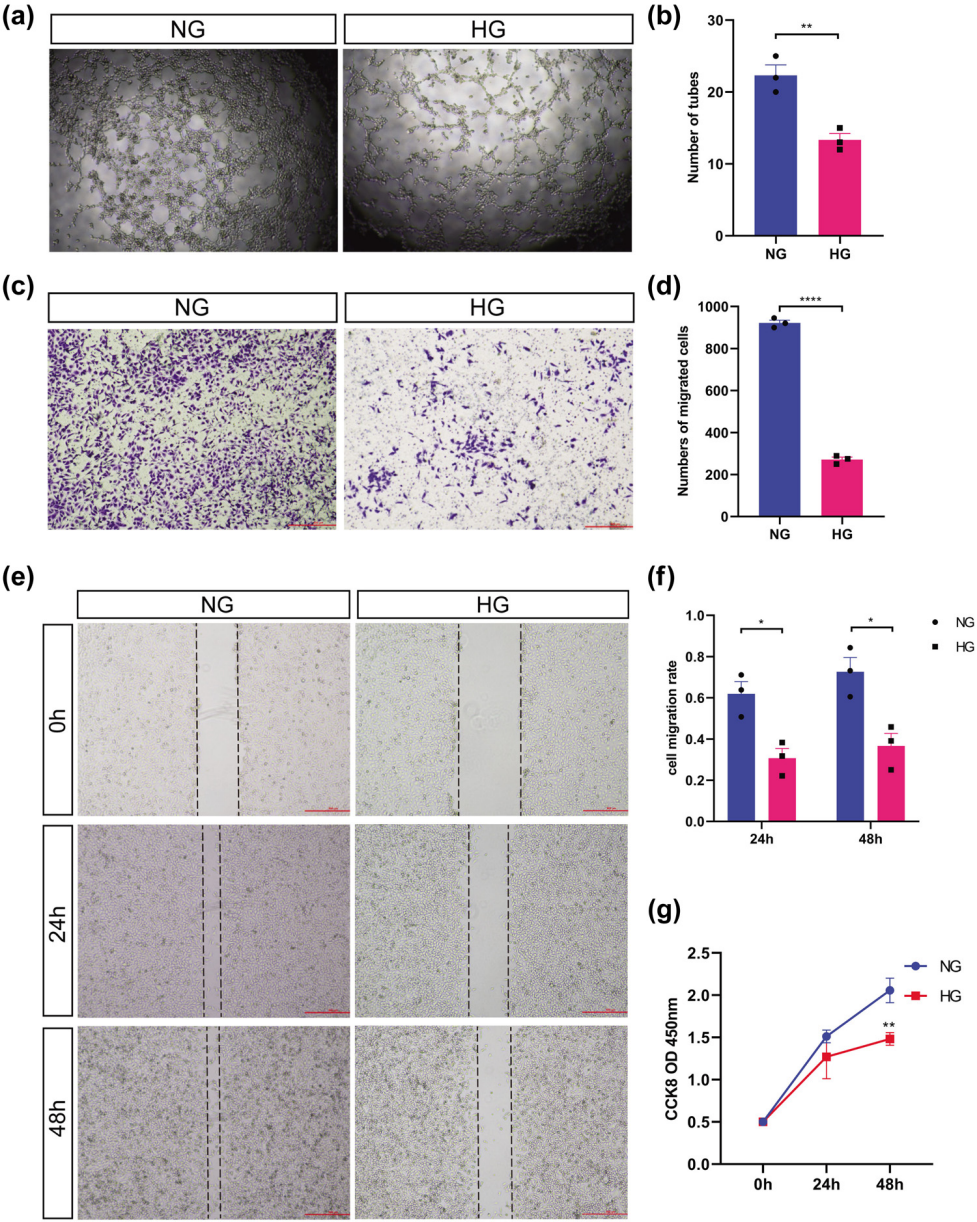
Effects of high glucose on proliferation, tube formation, and migration of HUVEC cells. (a) Tube formation (40×). (b) Quantitative analysis of the tube formation experiment. (c) Vertical migration (40×). (d) Quantitative analysis of the transwell assay. (e) Horizontal migration (40×). (f) Quantitative analysis of the wound healing experiment. (g) Proliferation. * *P* < 0.05, ** *P* < 0.01, and **** *P* < 0.0001. HG represents the high glucose stimulation group; NG represents the normal glucose concentration group.

HUVECs were stimulated with 30 mmol/L d-glucose for 24 and 48 h to establish high-glucose injury models, as previously reported [31,33,34]. The ability of HUVECs to form tubes, migrate, and proliferate after high glucose stimulation was detected. The results showed that high glucose inhibited the tube formation, vertical migration, horizontal migration, and proliferation of HUVECs (*P* < 0.05; Figure 5).

### 3.6 AQP8 overexpression inhibited the tube formation, migration, and proliferation of HUVEC cells

**Figure 6:**
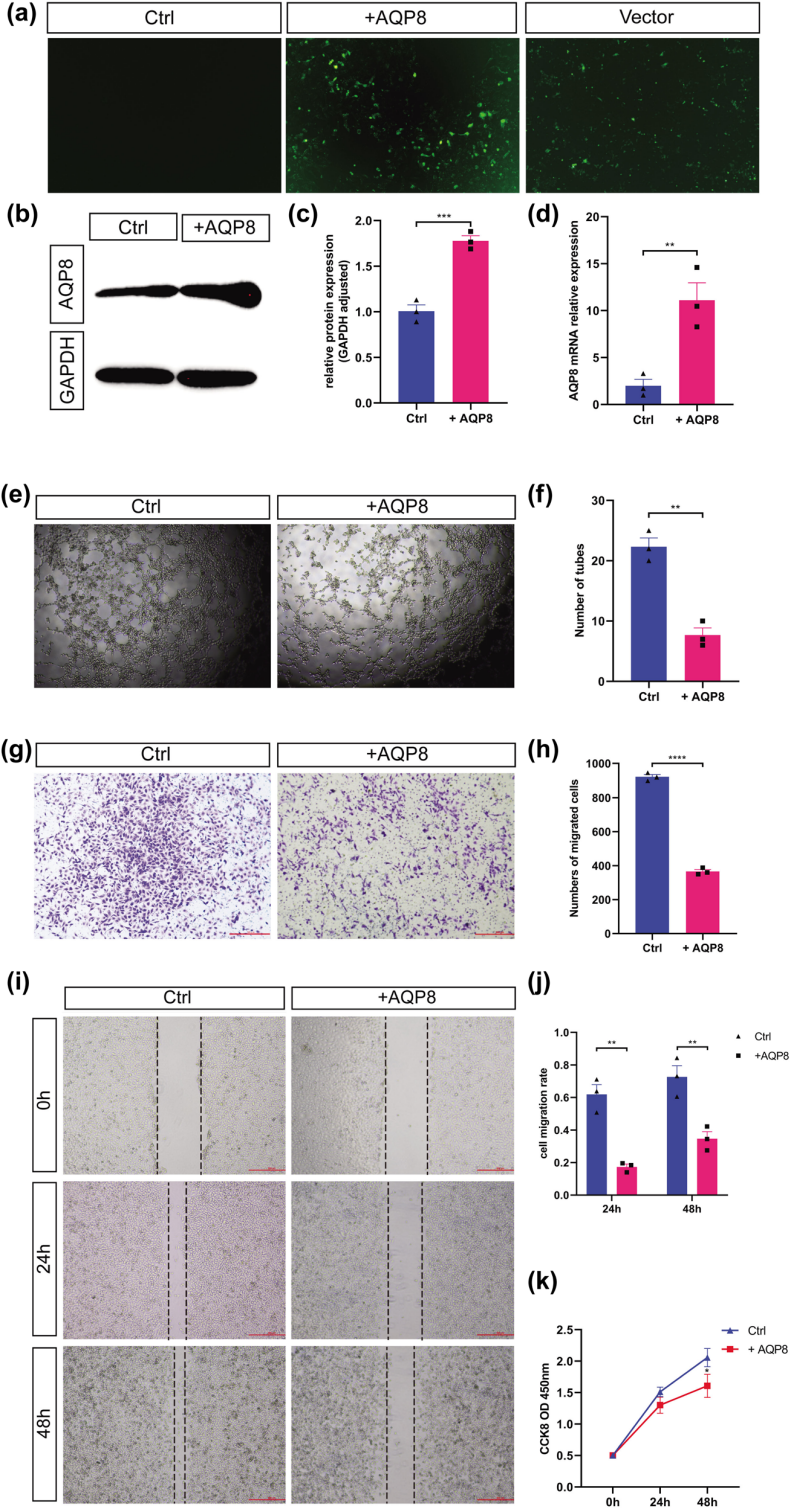
Effects of AQP8 overexpression on proliferation, tube formation, and migration of HUVEC cells. (a) Efficiency of plasmid transfection (40×). (b) Protein expression level of AQP8. (c) Quantitative analysis of the western blot analysis. (d) mRNA expression level of AQP8. (e) Tube formation (40×). (f) Quantitative analysis of tube formation. (g) Vertical migration (40×). (h) Quantitative analysis of the transwell assay. (i) Horizontal migration (40×). (j) Quantitative analysis of the wound healing experiment. (k) Proliferation. * *P* < 0.05, ** *P* < 0.01, *** *P* < 0.001, and **** *P* < 0.0001.

**Figure 7:**
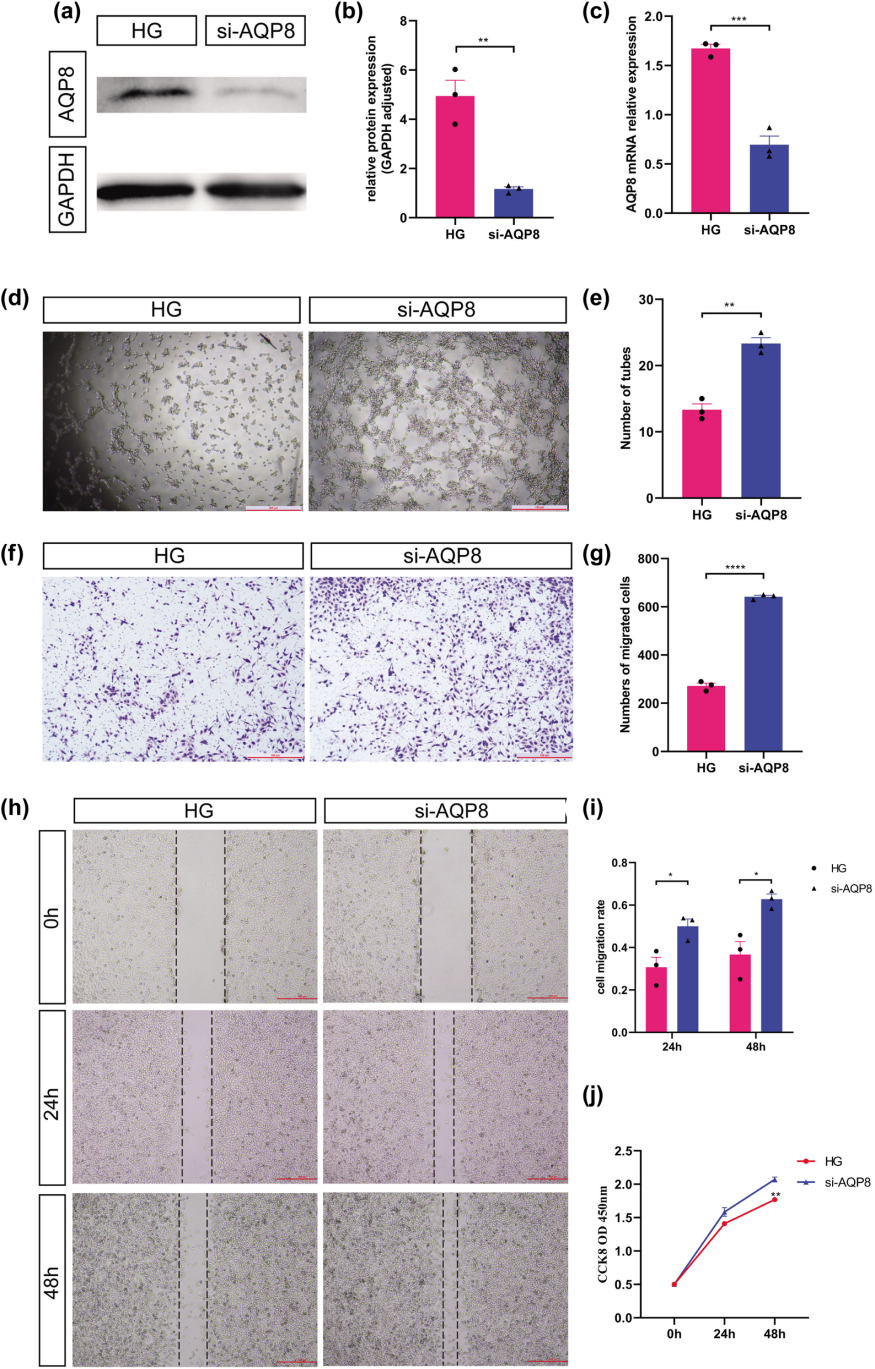
Downregulation of AQP8 to rescue the tube formation, migration, and proliferation changes after high glucose injury to HUVECs. (a) Protein expression level of AQP8 in HUVECs downregulated by si-AQP8 siRNA. (b) Quantitative analysis of the western blot analysis. (c) mRNA expression level of AQP8 in HUVECs transfected with si-AQP8 siRNA. (d) Tube formation (40×). (e) Quantitative analysis of tube formation experiment. (f) Vertical migration (40×). (g) Quantitative analysis of transwell assay. (h) Horizontal migration (40×). (i) Quantitative analysis of wound healing experiment. (j) Proliferation. * *P* < 0.05, ** *P* < 0.01, *** *P* < 0.001, and **** *P* < 0.0001. HG represents the high glucose stimulation group; NG represents the normal glucose concentration group.

To further understand the role and mechanism of AQP8 in placental vascular pathological changes, the effects of AQP8 on the behavior of HUVECs were analyzed. First, HUVECs were transfected with the AQP8 overexpression plasmid, and the transfection efficiency was detected after 48 h. As shown in Figure 6a, the AQP8 overexpression plasmid was successfully transfected into HUVECs. Western blotting and RT-qPCR were used to verify the transfection efficiency, and they showed that the expression levels of AQP8 protein and mRNA in the HUVECs were significantly increased after transfection with the AQP8 overexpression plasmid (all *P* < 0.05; Figure 6b–d). In addition, AQP8 overexpression significantly inhibited tube formation, vertical migration, horizontal migration, and proliferation of HUVECs (*P* < 0.05; Figure 6e–k). After high glucose stimulation, HUVECs were transfected with si-AQP8 to determine whether the behavior of the HUVECs could be reduced by knocking down AQP8. Western blotting and RT-qPCR were used to verify the transfection efficiency, and the results showed that AQP8 siRNA transfection effectively reduced the expression level of AQP8 in the HUVECs (all *P* < 0.05; Figure 7a–c). Moreover, downregulation of AQP8 significantly rescued tube formation, vertical migration, horizontal migration, and proliferation ability of HUVECs injured by high glucose stimulation (all *P* < 0.05; Figure 7d–j).

## 4 Discussion

In this study, the results showed that in the arterioles of the placentas of the GDM group, the number of microvessels significantly increased and the area of microvessels decreased, which is in accordance with the aforementioned studies. Evidence has shown that poor blood glucose control in GDM can lead to abnormal placental morphology [35,36,37]. In addition, changes in the vascular endothelial function may provide a pathological basis for placental vascular lesions in GDM [38]. The results of this study showed that the expression levels of VCAM-1, TNF-α, and VEGF-A were significantly increased in the GDM placentas. VCAM-1 is a biomarker of vascular endothelial dysfunction, and its expression levels in the maternal blood of pregnant women with GDM and umbilical cord blood are significantly higher than those in controls [39]. TNF-α is an inflammatory factor that can damage vascular endothelial cells by activating inflammatory signaling pathways. In GDM patients with vascular endothelial damage, TNF-α stimulates glomerular endothelial cells to express various adhesion factors, thus causing them to adhere to large numbers of neutrophils, lymphocytes, and monocytes. As neutrophils accumulate in large numbers locally, they release various inflammatory factors such as lysosomal enzymes, which hydrolyze the vascular basement membrane, inner elastic membrane, and connective tissue, thereby causing damage to blood vessels and their surrounding tissues [40,41]. In addition, inflammatory factors inhibit the activity of insulin receptor enzymes, phosphorylate receptor substrates, and respond to oxidative stress, resulting in impaired endothelial cell function [42]. High expression of TNF-α in GDM placental vascular endothelial cells can cause placental vascular endothelial dysfunction [43]. In addition, as the most important factor in regulating placental angiogenesis, VEGF can specifically stimulate the proliferation and migration of vascular endothelial cells and participate in the formation of new blood vessels by binding to the high-affinity tyrosine kinase receptor VEGF-R2, which affects the key link of vascular development [30]. The high expression of VCAM-1 and TNF-α in GDM placentas may be related to the disorder of placental vascular endothelial cells, and the increased expression of VEGF-A may be closely related to hyperplastic pathological neovascularization in placentas, which is consistent with the morphological changes of placentas in this study. These results suggest that the dysfunction of placental vascular endothelial cells in GDM may be an important reason for pathological changes in placental vascular endothelial cells.

Placental vascular injury in GDM may be closely related to hypoxia, hyperglycemia, insulin resistance, and other mechanisms, particularly hyperglycemia. In this study, we found that hyperglycemia plays a primary role in abnormal endothelial cell behavior. On the one hand, in GDM patients with hyperglycemia, the body produces more reactive oxygen species (ROS), which leads to an unbalanced oxidative stress response that causes placental vascular damage and even serious adverse pregnancy outcomes [44]. On the other hand, ROS activity can also be combined with hyperglycemia effects, thereby further leading to vascular damage, and this combined activity may be an important mechanism leading to placental vascular lesions in GDM [45,46]. Hyperglycemia has a significant effect on placental vascular injury, and HE staining revealed the morphological changes of microvessels in placental villi. HUVECs represent common vascular endothelial cells and can simulate the high-glucose microenvironment of GDM to study the effect of high glucose on the behavior of vascular endothelial cells. Thus, we established a high-glucose model of HUVEC to simulate the high-glucose microenvironment of GDM placental vascular endothelial cells and further explored the effect of high glucose on the behavior of endothelial cells. Chen et al. found that a high glucose concentration of 30 mmol/L could induce HUVEC injury, which was not affected by osmotic pressure [47]. Thus, in this study, 30 mmol/L d-glucose was used to stimulate HUVECs. After high glucose stimulation for 24 and 48 h, the expression levels of VCAM-1, TNF-α, and VEGF-A were significantly higher than that of the normal control group. These results showed that high glucose levels could damage vascular endothelial cells, thus leading to high expression of VCAM-1, TNF-α, and VEGF-A. In a high-glucose environment, HUVECs produce more ROS, and an abnormal increase in ROS leads to the destruction of lipids, DNA, and proteins, resulting in abnormal cell function, such as inhibiting cell proliferation, migration, and invasion, and increasing apoptosis [48]. The results also showed that the proliferation, migration, and tube-forming ability of HUVECs decreased after high glucose stimulation. In addition, with the high expression of VEGF-A, the effect of high glucose on vascular injury was more obvious, which significantly inhibited the behavior of HUVEC. Therefore, this study suggests that the expression of endothelial dysfunction markers, including VCAM-1, TNF-α, and VEGF-A, was increased in high glucose-stimulated HUVEC, leading to dysfunction of vascular endothelial cells in tube formation, migration, and proliferation and pathological changes in the placental vascular structure.

AQPs belong to the main endogenous protein superfamily and function as membrane channels for selective water transport [17]. Recent studies have shown that AQPs can be detected in vascular endothelial cells and are involved in angiogenesis, cell migration, adhesion, and other biological processes [24]. However, to date, few studies have reported the relationship between AQP8 and hematopoiesis or vascular development. In this study, we found that AQP8 was localized in placental vascular endothelial cells and further confirmed that AQP8 was highly expressed in GDM placentas. To determine whether AQP8 affects the pathological changes of placental vascular endothelial cells and even placental vessels in GDM, HUVECs were stimulated by high glucose, and the results showed that the expression of AQP8 significantly increased after HUVECs were injured by high glucose, which indicated that AQP8 might participate in the dysfunction of HUVECs through the regulation of related signaling pathways, and then change the vascular structure of GDM placentas. To further verify the effect of AQP8 on the behavior of endothelial cells, we transfected an AQP8 overexpression plasmid into HUVECs and found that high expression of AQP8 could inhibit the proliferation, tube formation, and migration of HUVECs. However, after the behavior of HUVECs was damaged by high glucose, the proliferation, tubulation, and migration of HUVECs could be reduced by downregulating the expression of AQP8 by transfecting AQP8 siRNA. These results further suggested that AQP8 can cause vascular endothelial cell dysfunction and GDM placental vascular damage, thus leading to GDM placental vascular lesions.

This study reported that AQP8 may be involved in changes in placental vascular endothelial cell behavior and placental vascular structure through the regulation of related signaling pathways. However, this study has several limitations. First, the specific mechanisms need to be further studied. Second, the mode of delivery was not differentiated; therefore, it was not possible to determine whether the mode of delivery had an impact on the results of this study. Third, the study period must be extended, and the sample size must be increased in future studies.

## 5 Conclusion

High glucose could increase the expression of endothelial dysfunction markers of VCAM-1, TNF-α, and VEGF-A, thus leading to the dysfunction of vascular endothelial cells in terms of tube formation, migration, and proliferation and pathological changes in the placental vascular structure. Upregulated expression of AQP8 may be associated with the abnormal behavior of vascular endothelial cells and vascular pathological changes in GDM placentas.
